# ART Innovations: Fostering Women’s Psychophysical Health between Bioethics Precepts and Human Rights

**DOI:** 10.3390/healthcare9111486

**Published:** 2021-11-01

**Authors:** Simona Zaami, Lorenza Driul, Milena Sansone, Elisa Scatena, Karin Louise Andersson, Enrico Marinelli

**Affiliations:** 1 Department of Anatomical, Histological, Medicolegal and Orthopedic Sciences, Sapienza University of Rome, 00161 Rome, Italy; enrico.marinelli@uniroma1.it; 2 Clinic of Obstetrics and Gynecology, Hospital of Udine, DAME, University of Udine, 33100 Udine, Italy; lorenza.driul@uniud.it; 3 Department of Obstetrics and Gynecology, Sant’Eugenio Hospital, 00144 Rome, Italy; milena.sansone@aslroma2.it; 4 Department of Gynecology and Obstetrics, Santo Stefano Hospital, 59100 Prato, Italy; elisa1.scatena@uslcentro.toscana.it; 5 Department of Territory Health, Azienda Sanitaria Toscana Centro, 50012 Florence, Italy; karinlouise.andersson@uslcentro.toscana.it

**Keywords:** assisted reproductive technologies (ART), female infertility, procreative rights, psychophysical well-being

## Abstract

Infertility is a highly relevant global issue affecting the reproductive health of at least 15% of reproductive-aged couples worldwide. The scope and severity of the infertility problem is even more prevalent in developing countries, mostly due to untreated reproductive tract infections (RTIs). Infertility, however, goes beyond the mere inability to procreate, but brings about profound psychological, social, and ethical implications of enormous magnitude. In vitro fertilization (IVF) and other assisted reproduction technologies (ARTs) have gradually become widespread therapeutic options. After all, the implementation of medically assisted reproductive procedures in order to overcome infertility is in keeping with the tenets of the reproductive rights agenda laid out at the International Conference on Population and Development (ICPD) in Cairo in 1994. Nonetheless, concerns still linger about how to implement and regulate such interventions in an ethically tenable fashion. The unremitting pace at which such techniques develop have upset the very notion of sexuality relating to reproduction as well as the concept of family itself. That rift risks causing a crisis in terms of bioethics sustainability and enforcement, which is bound to happen when science and innovation outpace the bioethical precepts on which we rely for essential guidance in medical practice. The authors argue in favor of an approach to regulation and policy-making that puts on the forefront a thorough assessment as to potential risks that such interventions might entail for foundational bioethics principles and inalienable human rights.

## 1. Introduction

Infertility is a major factor jeopardizing the reproductive health of at least 15% of reproductive-aged couples worldwide, which undoubtedly makes it a highly relevant global issue. As for female infertility, the main focus of this writing, 9% of reproductive-aged women, including nearly 1.5 million women in the United States, are reportedly infertile [1]. The scope and severity of the infertility problem is even more prevalent in developing countries, mostly due to untreated reproductive tract infections (RTIs) [2]. Female infertility can be brought about by various underlying conditions, and it may be ascribed to uterine, vaginal, endocrine, cervical, tubal, and pelvic-peritoneal determining factors. In addition, research shows how the etiology of a sizeable share of infertility cases, as many as 15–30% overall, is still unidentified [3]. It would be shallow to view infertility as a merely functional limitation: the inability to procreate has, in fact, far-reaching ramifications that ought to be weighed thoroughly in order to gain a full understanding as to real scope of the problem. It is well beyond the realm of parenthood in and of itself, since the social, psychological, and even economic implications have been broadly researched. Undoubtedly, female infertility, along with related diagnoses, have repercussions deeply affecting overall health. In addition to meeting the patients’ immediate reproductive needs in terms of treatment, the broader health impact stemming from all the specific causes of infertility must not be neglected by healthcare professionals, in order to provide accurate counselling pertaining to long-term risk and individually tailored therapeutic approaches. The authors have set out to briefly expound upon the complexities inherent in the relationship between fertility and health (whether physical or mental), to figure out a tenable balance between fundamental rights, bioethics precepts and legal/regulatory approaches. This may well be the only way to outline a path ahead towards more effective safeguards for those who strive to achieve parenthood in the face of a condition of infertility.

## 2. The Mental and Emotional Toll of Female Infertility

To many individuals and couples, infertility is no less than a life crisis triggering severe emotional distress, and patients receiving assisted reproductive therapies risk incurring psychiatric comorbidity [4]. Infertile patients are likely to incur a higher risk of anxiety, frustration, hopelessness, guilt, depression, diminished self-esteem, and personal worthlessness. In addition, spousal issues often arise within the couple, commonly due to the pressure in making consequential treatment decisions and outcomes [5]. The prevalence of such disorders appear to be rather significant among infertile women: research findings show that 40% of that segment meet the criteria for a psychiatric diagnosis. The most widespread diagnosis points to anxiety disorders, major depressive disorder and dysthymia [6]. An increased risk of suicidal ideation has been reported in women who suffer from infertility (e.g., a 9.4% incidence), although a direct correlation is still undetermined [7]. It is hardly surprising that an even higher risk has been reported for women whose attempts to get pregnant through medically-assisted procreation (MAP) procedures were unsuccessful [8]. It is quite worrisome to observe that, notwithstanding such substantial rates of psychiatric comorbidity, relatively few women resort to psychiatric care (a study indicates as few as 6.7% [9]). Overall, research has estimated the incidence of psychological and emotional issues for infertile couples to be as high as 25–60% [10,11]. Such a high degree of variability is mostly due to the complex set of variables that play a role in the condition, e.g., gender, cause and duration of infertility, treatment options. The incidence of depression and anxiety in infertile couples has been found to be substantially higher than in fertile counterparts and in the general population, respectively. Obsessive-compulsive signs translating into psychoticism, substance abuse, and eating disorders have also been associated with infertility. Women have been found to be more profoundly impacted than men [12]. At the same time, it is worth noting that mental and psychological distress and psychiatric conditions have been linked to infertility treatment as well. For instance, the 2-week time span separating the embryo transfer from the pregnancy test is known to be considerably stressful, and the potential consequences of symptoms on MAP outcome and pregnancy rates has been explored, though no decisive evidence to make such connections has been produced so far; women should therefore be reassured that there is no evidence that psychological or psychiatric conditions caused by or worsened by fertility issues will compromise the possibility of achieving motherhood [13]. Nonetheless, assisted reproduction procedures are without a doubt emotionally demanding, and patients who struggle to adapt must be identified early [14]. Research findings show that women undergoing infertility treatment and MAP procedures are substantially more likely to screen positive for major depressive disorder, anxiety or both [15,16,17]. It is of utmost importance that healthcare operators catering to women with fertility issues are fully aware of their patients’ emotional and mental responses, so that possible psychiatric morbidity after fertility problems can be identified in a timely fashion and treated. Although no formal requirements are currently in place for providing psychological counselling for infertility patients, including psychological support into MAP practice is recognized as potentially beneficial. The high levels of stress caused by the condition of infertility have been well documented. Even though the impact of stress on fertility treatment outcomes is still rather controversial, it is likely that psychological interventions for women with infertility have the potential to allay anxiety and depressive symptoms to some degree, thus contributing to more favorable outcomes and higher pregnancy rates [18]. That being said, more research is needed on that point, given that the numerous studies assessing the efficacy of psychological interventions on women with infertility have not agreed on the results [19,20,21].

## 3. Women in Developing Countries Are Hardest Hit

As mentioned earlier, the burden of infertility is even more severe among women living in low-income countries, which has to do with broad-ranging socioeconomic and cultural implications. In such areas of the world, already plagued by disproprtionately high mortality rates [22], having biological children is expected of couples and highly valued in terms of socioeconomic status. Hence, infertility leading to involuntary childlessness is stigmatized, resulting in public shame and humiliation, social isolation and diminished or lost status, economic deprivation, and even violence. Ever since the 1990s, research has shown that high rates of infertility and ensuing childlessness are among the most relevant and underestimated reproductive health issues in developing countries [23,24,25]. The inability to have children is very often viewed as no less than a personal tragedy—a real curse for the family, with dire implications that may go beyond the couple and affect the local community. In addition, the failure to procreate frequently gives rise to psychosocial repercussions of considerable severity [26,27]. That is largely due to the fact that womanhood is inextricably bound to motherhood, hence women suffering from infertility are often stigmatized and blamed for the couple’s failure to have an offspring. Social elements should not be overlooked either: the elderly are generally economically dependent on their children because of the absence or inadequacy of social security systems. Childless women are frequently marginalized and abused, resulting in isolation, neglect, domestic violence, and polygamy. Worryingly, infertility can affect as many as 30% of reproductive-aged women in some regions of Sub-Saharan and Northern Africa, the Middle East, South and Central Asia, and Eastern Europe.

Bilateral tubal occlusion, typically caused by sexually transmitted diseases and pregnancy-related infections, has been reported to be the number one cause of infertility in developing countries [28]; although such a condition is treatable with assisted reproductive technologies (ART), in developing countries, such procedures are either unavailable or so costly as to be inaccessible for most women. Further exacerbating that already critical scenario, countless women in developing countries have no chance to access quality maternal care to exercise their reproductive rights. Nonetheless, as far as the enforcement of such rights is concerned, it is worth remarking that, since the International Conference on Population and Development (ICPD) was approved in 1994, relevant steps have been taken by states towards better maternal health outcomes overall. The recognition of the human rights obligations of states has been highlighted in the Alyne vs. Brazil case, brought before the United Nations Committee on the Elimination of Discrimination against Women (CEDAW, the body in charge of monitoring the implementation of the Convention on the Elimination of All Forms of Discrimination against Women [29]) in 2007 by the Center for Reproductive Rights [30]. Alyne was a 28-year-old Afro-Brazilian woman who perished from pregnancy complications after being denied quality maternal healthcare by both a private and a public health facility. In light of the facts, and given how wantonly and brutally Alyne’s reproductive rights had been violated, the CEDAW Committee affirmed that States have an immediate and enforceable obligation to address and reduce maternal mortality, and that quality maternal healthcare must be provided to all women, free from discrimination—regardless of race, income, or geographic location. Such human rights obligations in the Alyne case have been acknowledged by courts of law at the national level as well, notably in the case Josephine Majani vs. Attorney General of Kenya. Although Kenya has put in place a universal maternal healthcare system, adequate care is not available for countless women poor women who can only access often saturated and inadequate government facilities. Ms. Majni was allegedly beaten and disrespected after she passed out while giving birth on the floor. No bed or professional assistance was ever made available to her. The Nairobi High Court’s decision unequivocally pointed out the absolute urgency of bringing down maternal morbidity and mortality, while making access to free and respectful obstetric care available for those in need. Those have to be viewed as cornerstones of an ongoing effort to uphold the rights to health and non-discrimination, along with the drafting of adequate modern standards and recommendations for healthcare professionals and facilities [31]. 

## 4. Medically-Assisted Procreation as an Essential Tool for Upholding Reproductive Rights and Autonomy

As we have attempted to briefly expound upon, there is no denying that medically assisted procreation based on assisted reproductive technologies has tremendous potential, not only in terms of enabling women to procreate who otherwise could not: the inability to procreate has, in fact, psychological, social, economic, and family repercussions of great magnitude.

MAP certainly constitutes an extremely valuable tool for enabling millions of women to fulfill their procreative potential and exercise their right to procreative autonomy, for the ultimate purpose of achieving better health and quality of life overall. At the same time, it would be remiss to overlook the moral, ethical, legal, and regulatory complexities which such practices entail. Although it is undetermined whether infertility in itself can affect a patient’s “physical health”, and because establishing a causal relationship is therefore not currently feasible, the very notion of health appears to be broader than mere absence of disease. The World Health Organization defines “health” as a state not only of physical well-being but mental and social well-being as well [32]. According to that line of reasoning, the ICPD has characterized reproductive health as a condition presenting complete physical, mental, and social well-being, hence not merely the absence of illness, in all aspects relative to the reproductive system, its functional viability, and all related processes. The notion of reproductive health should therefore be construed as implying that people have the full capability to conceive and reproduce, as well as the freedom to decide if, when, and how often to do so. The right to have information and access to appropriate healthcare services that provides couples with the best chance of having a healthy infant and favorable maternal outcomes is intended as implicit in that condition [33].

As we have elaborated earlier on, infertility can jeopardize and greatly harm mental or social well-being by causing psychological distress and affliction, and millions of individuals every year willingly bear considerable physical and financial costs in order to overcome it. Still, many countries that do provide universal healthcare for medically essential medical intervention and treatments do not fund ART, although they generally provide funding for diagnostic tests and even pharmacological and surgical treatments that restore fertility. That is because ART overcomes the condition of infertility by artificial means of conception, rather than medically reversing it. Such dynamics raise daunting ethical quandaries: in light of the core human rights principle of non-discrimination on disability grounds, should states ethically do more than to merely allow those who can afford it to use ART services? Should ART be allowed as a sort of “luxury medicine”, not unlike cosmetic surgery, available to those who can pay for it, or does the principle of equity require at least some degree of public funding of ART services, in order to make it accessible to as many infertile patients as possible? Although such questions do need answers, in the interest of the millions of women afflicted by infertility, the funding of ART is only part of a much broader problem. 

MAP technologies have in fact developed at such a pace that they have revolutionized the very notion of procreation, making it possible for couples and singles to have children well beyond their “natural” capabilities [34]. Procreation has become disjointed from sexuality [35]. Women suffering from absolute uterine-factor infertility, either congenital or arising from previous pregnancy complications [36,37], and even same-sex couples, can nowadays achieve parenthood and have genetically related offspring. Practices such as gamete donation, oocyte and embryo freezing, surrogacy, uterine transplant [38] have profoundly upset the genetic cohesion and integrity of traditional family identity, and have often elicited conservative responses, particularly in countries with deep-rooted Catholic tradition such as Italy. Legislative responses have therefore tended to mount some sort of defence against supposed risks to traditional moral values, at times more instinctive than scientific or intellectual [39]. Countries, not unlike individuals, may vary considerably in the way they ascribe value and safeguards to embryos or fetuses, or how they perceive the nature of family, the burdens on infertile couples, and all the other aspects and interests at stake in ART and the way in which it is governed and restricted [40,41]. While each country is free to set up legislative frameworks that best reflect their cultural distinctive traits and social priorities, it is essential to make sure that a common foundational ethical and moral bedrock is preserved and upheld [42]. European Court of Human Rights decisions have repeatedly asserted the wide margin of appreciation granted to member states on moral issues, particularly when there is no European consensus. Nonetheless, it is incumbent upon the states to substantiate and justify the decisions and approaches they choose to undertake, in order to strike a fair balance between competing private and public interests and reconcile the legitimate hopes, needs, desires, and aspirations of all those involved [43,44]. That is to be viewed as part and parcel of the broader duty each country has committed to fulfilling, via the signing of international treaties, in regards to civil and human rights enforcement [45,46,47]. The very constitution of the WHO itself asserts beyond the shadow of a doubt that “the enjoyment of the highest attainable standard of health is a fundamental right of every human being without distinction of race, religion, political belief, economic or social condition,” and that national governments must be held responsible “for the health of their peoples which can be fulfilled only by the provision of adequate health and social measures.” [48]. Just as meaningfully, that very concept is buttressed in article 25.1 of the Universal Declaration of Human Rights (UDHR), which again stresses state responsibilities in relation to health and medical care, by arguing that “everyone has the right to a standard of living adequate for the health and well-being of himself and of his family, including food, clothing, housing and medical care and necessary social services.” [49]. Still, those broad-ranging assertions need to be constantly contextualized, if they are to retain their effectiveness: scientific technologies and advancements are bound to broaden the horizons of reproductive technologies, among many other areas of research, hence the broader notion of health, well-being and self-fulfillment, which some believe includes the full exercise of reproductive rights that are constantly evolving [50]. The medical community, legislators and policy-makers need to take stock as to where we are headed, since there is no denying that such practices are bound to further evolve and in the not-so-distant future, and the biological paradigm underlying current gender categories could be further upset as reproductive functions are carried out through increasingly artificial means, such as ectogenesis [51]. An issue of increasing relevance and high complexity is, for instance, ART access, particularly fertility preservation, for transsexual individuals about to be reassigned. It is not known how the psychological effects may play out for such patients, hence it is extraordinarily hard to draw up a set of good practices to guide professionals in such situations.

## 5. Conclusions: Procreative Revolution and Harmonized Standards for the Sake of Equitable Access

Certainly, reproductive technologies have outpaced the cultural, moral, and ethical evolution of our societies, and legislative responses reflect such discrepancies. MAP procedures have in fact been regulated with varying degrees of restrictions in Europe and worldwide, and that has worsened the inequalities between those who can afford “fertility travels” to countries with permissive legislation and those who cannot, thus failing to uphold the latter’s reproductive rights. Taking into account the European Union, that is largely due to the fact that the European Court of Human Rights affords a broad margin of appreciation to member states in matters involving social, moral, and ethical values. Nevertheless, there is widespread agreement that the welfare of the child should be applied as a majorly important measurement when it comes to granting access to medically assisted reproduction. At the same time, the way in which this criterion is made operational differs widely. That is due to a large degree by the fact that varying evaluation criteria are applied. However difficult, it is essential that the highest possible degree of legislative and regulatory harmonization ought to be pursued, at least among countries such as EU members, which share a common set of fundamental core values. Practices involving the commodification and sale of human reproductive cells and embryos ought to be punished as criminal offences, as must be the exploitation and coercion of women and men victimized for reproductive trafficking, particularly in inter-country MAP and surrogacy procedures. Any violation of principles such as equity, justice, and respect for human dignity and inalienable human rights, irrespective of the victim’s alleged “consent”, must be criminalized and punished wherever it has taken place. Ultimately, it is extremely challenging to strike a morally and ethically tenable balance between the rights of different parties, which can at times be conflicting. Providing alternatives to procreative travelling, which also poses an element of discrimination between those who can financially resort to it and those who cannot, is a reasonably balanced starting point. To that end, relying on common, widely shared guidance is the only way to ensure that all people who seek to achieve parenthood have a reasonable chance to access treatments through which they can exercise their fundamental right to procreate. 

## Data Availability

The data presented in this study are available on request from the corresponding author.

## References

[1] Tsevat D.G., Wiesenfeld H.C., Parks C., Peipert J.F. (2017). Sexually Transmitted Diseases and Infertility. Am. J. Obstet. Gynecol..

[2] Rouchou B. (2013). Consequences of Infertility in Developing Countries. Perspect. Public Health.

[3] Gunn D.D., Bates G.W. (2016). Evidence-Based Approach to Unexplained Infertility: A Systematic Review. Fertil. Steril..

[4] Rooney K.L., Domar A.D. (2018). The Relationship between Stress and Infertility. Dialog. Clin. Neurosci..

[5] Baghianimoghadam M.H., Aminian A.H., Baghianimoghadam B., Ghasemi N., Abdoli A.M., Seighal Ardakani N., Fallahzadeh H. (2013). Mental Health Status of Infertile Couples Based on Treatment Outcome. Iran. J. Reprod. Med..

[6] Becker M.A., Chandy A., Mayer J.L.W., Sachdeva J., Albertini E.S., Sham C., Worley L.L.M. (2019). Psychiatric Aspects of Infertility. Am. J. Psychiatry.

[7] Shani C., Yelena S., Reut B.K., Adrian S., Sami H. (2016). Suicidal Risk among Infertile Women Undergoing In-Vitro Fertilization: Incidence and Risk Factors. Psychiatry Res..

[8] Kjaer T.K., Jensen A., Dalton S.O., Johansen C., Schmiedel S., Kjaer S.K. (2011). Suicide in Danish Women Evaluated for Fertility Problems. Hum. Reprod..

[9] Chen T.-H., Chang S.-P., Tsai C.-F., Juang K.-D. (2004). Prevalence of Depressive and Anxiety Disorders in an Assisted Reproductive Technique Clinic. Hum. Reprod..

[10] Lechner L., Bolman C., van Dalen A. (2007). Definite Involuntary Childlessness: Associations between Coping, Social Support and Psychological Distress. Hum. Reprod..

[11] Hasanpoor-Azghdy S.B., Simbar M., Vedadhir A. (2014). The Emotional-Psychological Consequences of Infertility among Infertile Women Seeking Treatment: Results of a Qualitative Study. Iran. J. Reprod. Med..

[12] De Berardis D., Mazza M., Marini S., Del Nibletto L., Serroni N., Pino M.C., Valchera A., Ortolani C., Ciarrocchi F., Martinotti G. (2014). Psychopathology, Emotional Aspects and Psychological Counselling in Infertility: A Review. Clin. Ter..

[13] Boivin J., Griffiths E., Venetis C.A. (2011). Emotional Distress in Infertile Women and Failure of Assisted Reproductive Technologies: Meta-Analysis of Prospective Psychosocial Studies. BMJ.

[14] Boivin J. (2019). How Does Stress, Depression and Anxiety Affect Patients Undergoing Treatment?. Curr. Opin. Obstet. Gynecol..

[15] Sejbaek C.S., Hageman I., Pinborg A., Hougaard C.O., Schmidt L. (2013). Incidence of Depression and Influence of Depression on the Number of Treatment Cycles and Births in a National Cohort of 42,880 Women Treated with ART. Hum. Reprod..

[16] Pasch L.A., Holley S.R., Bleil M.E., Shehab D., Katz P.P., Adler N.E. (2016). Addressing the Needs of Fertility Treatment Patients and Their Partners: Are They Informed of and Do They Receive Mental Health Services?. Fertil. Steril..

[17] Lakatos E., Szigeti J.F., Ujma P.P., Sexty R., Balog P. (2017). Anxiety and Depression among Infertile Women: A Cross-Sectional Survey from Hungary. BMC Womens Health.

[18] Ying L., Wu L.H., Loke A.Y. (2016). The Effects of Psychosocial Interventions on the Mental Health, Pregnancy Rates, and Marital Function of Infertile Couples Undergoing in Vitro Fertilization: A Systematic Review. J. Assist. Reprod. Genet..

[19] Boivin J. (2003). A Review of Psychosocial Interventions in Infertility. Soc. Sci. Med..

[20] Hämmerli K., Znoj H., Barth J. (2009). The Efficacy of Psychological Interventions for Infertile Patients: A Meta-Analysis Examining Mental Health and Pregnancy Rate. Hum. Reprod. Update.

[21] Chow K.-M., Cheung M.-C., Cheung I.K. (2016). Psychosocial Interventions for Infertile Couples: A Critical Review. J. Clin. Nurs..

[22] World Health Organization Maternal Mortality. Issued on 19th September 2019. https://www.who.int/news-room/fact-sheets/detail/maternal-mortality.

[23] Bergström S. (1992). Reproductive Failure as a Health Priority in the Third World: A Review. East Afr. Med. J..

[24] Leke R.J., Oduma J.A., Bassol-Mayagoitia S., Bacha A.M., Grigor K.M. (1993). Regional and Geographical Variations in Infertility: Effects of Environmental, Cultural, and Socioeconomic Factors. Environ. Health Perspect..

[25] Ombelet W., Cooke I., Dyer S., Serour G., Devroey P. (2008). Infertility and the Provision of Infertility Medical Services in Developing Countries. Hum. Reprod. Update.

[26] Dyer S.J. (2007). The Value of Children in African Countries: Insights from Studies on Infertility. J. Psychosom. Obstet. Gynaecol..

[27] Umezulike A.C., Efetie E.R. (2004). The Psychological Trauma of Infertility in Nigeria. Int. J. Gynaecol. Obstet..

[28] Inhorn M.C., Patrizio P. (2015). Infertility around the Globe: New Thinking on Gender, Reproductive Technologies and Global Movements in the 21st Century. Hum. Reprod. Update.

[29] United Nations The Convention on the Elimination of All Forms of Discrimination against Women (CEDAW), Adopted in 1979 by the UN General Assembly. https://www.un.org/womenwatch/daw/cedaw/text/econvention.htm.

[30] Center for Reproductive Rights Human Rights and ICPD+25. Issued in August 2019. https://reproductiverights.org/sites/default/files/2019-09/GLP-ICPD25-FS-Web.pdf.

[31] Brown R., Kismödi E., Khosla R., Malla S., Asuagbor L., Andión-Ibanez X., Gruskin S. (2019). A Sexual and Reproductive Health and Rights Journey: From Cairo to the Present. Sex. Reprod. Health Matters.

[32] Larson J.S. (1996). The World Health Organization’s Definition of Health: Social versus Spiritual Health. Soc. Indic. Res..

[33] United Nations Programme of Action, 7.2 (U.N. Doc. A/CONF.171/13/Rev. 1). Proceedings of the United Nations International Conference on Population and Development.

[34] Klipstein S. (2012). Ethical Considerations Arising from the Use of Assisted Reproductive Technologies. Semin. Reprod. Med..

[35] Benagiano G., Carrara S., Filippi V. (2010). Sex and Reproduction: An Evolving Relationship. Hum. Reprod. Update.

[36] Zaami S., Montanari Vergallo G., Napoletano S., Signore F., Marinelli E. (2018). The Issue of Delivery Room Infections in the Italian Law. A Brief Comparative Study with English and French Jurisprudence. J. Matern. Fetal Neonatal Med..

[37] Valentini A.L., Gui B., Ninivaggi V., Miccò M., Giuliani M., Russo L., Marini M.G., Tintoni M., Cavaliere A.F., Bonomo L. (2017). The Morbidly Adherent Placenta: When and What Association of Signs Can Improve MRI Diagnosis? Our Experience. Diagn. Interv. Radiol..

[38] Zaami S., Marinelli E., di Luca N.M., Montanari Vergallo G. (2017). Ethical and Medico-Legal Remarks on Uterus Transplantation: May It Solve Uterine Factor Infertility?. Eur. Rev. Med. Pharmacol. Sci..

[39] Sallam H.N., Sallam N.H. (2016). Religious Aspects of Assisted Reproduction. Facts Views Vis. Obgyn..

[40] Londra L., Wallach E., Zhao Y. (2014). Assisted Reproduction: Ethical and Legal Issues. Semin. Fetal Neonatal Med..

[41] Shalev C., Moreno A., Eyal H., Leibel M., Schuz R., Eldar-Geva T. (2016). Ethics and Regulation of Inter-Country Medically Assisted Reproduction: A Call for Action. Isr. J. Health Policy Res..

[42] Harper J.C., Geraedts J., Borry P., Cornel M.C., Dondorp W., Gianaroli L., Harton G., Milachich T., Kääriäinen H., Liebaers I. (2013). Current Issues in Medically Assisted Reproduction and Genetics in Europe: Research, Clinical Practice, Ethics, Legal Issues and Policy. European Society of Human Genetics and European Society of Human Reproduction and Embryology. Eur. J. Hum. Genet..

[43] Robertson J.A. (2004). Protecting Embryos and Burdening Women: Assisted Reproduction in Italy. Hum. Reprod..

[44] Harper J.C., Aittomäki K., Borry P., Cornel M.C., de Wert G., Dondorp W., Geraedts J., Gianaroli L., Ketterson K., Liebaers I. (2018). Recent Developments in Genetics and Medically Assisted Reproduction: From Research to Clinical Applications. Eur. J. Hum. Genet..

[45] de Wert G., van der Hout S., Goddijn M., Vassena R., Frith L., Vermeulen N., Eichenlaub-Ritter U., ESHRE Ethics Committee (2021). The Ethics of Preconception Expanded Carrier Screening in Patients Seeking Assisted Reproduction. Hum. Reprod. Open.

[46] Nikogosian H., Kickbusch I. (2016). The Legal Strength of International Health Instruments—What It Brings to Global Health Governance?. Int. J. Health Policy Manag..

[47] Forman L. (2018). Human Rights Treaties Are an Important Part of the “International Health Instrumentariam” Comment on “The Legal Strength of International Health Instruments—What It Brings to Global Health Governance?”. Int. J. Health Policy Manag..

[48] World Health Organization (1946). Constitution of the World Health Organization. Am. J. Public Health Nations Health.

[49] United Nations General Assembly Universal Declaration of Human Rights. Adopted by the United Nations General Assembly of Its 183rd Meeting, Held in Paris on 10 December 1948. https://www.ohchr.org/EN/UDHR/Documents/UDHR_Translations/eng.pdf.

[50] Turner J.V., McLindon L.A. (2018). Bioethical and Moral Perspectives in Human Reproductive Medicine. Linacre Q..

[51] MacKay K. (2020). The “Tyranny of Reproduction”: Could Ectogenesis Further Women’s Liberation?. Bioethics.

